# Response to: Is it time to rethink the MCCQE Part II?

**Published:** 2016-03-31

**Authors:** M. Ian Bowmer

**Affiliations:** Executive Director and CEO, Medical Council of Canada

The challenging letter from Lougheed allows us an opportunity to review the history and purpose of the Medical Council of Canada Qualifying Examination (MCCQE) Part 2. The Medical Council of Canada (MCC) was established in 1912 to provide a qualification for licensure acceptable to all Canada’s medical regulatory authorities (MRAs). Under Council leadership, Canada became the first country in the world to administer a national licensing examination. MCC has a unique governance structure comprised of registrars and representatives from each of the provinces’ and territories’ MRAs, a senior faculty representative from each of the 17 medical schools, five public members and members representing students and residents. As a consequence, Council initiatives represent the deliberations and will of a broad group of stakeholders, especially the medical regulatory authorities.

Over the past hundred years the number of medical schools has grown considerably and with it, diversity has increased. Despite common accreditation standards, each of Canada’s 17 Faculties of Medicine is unique as they respond to regional differences, acknowledge local social accountabilities and maximize local resources to meet their educational mission. Medical school differences are even greater in other countries such as the UK, as described by Swanson and Roberts.^1^ Today, more than ever, medical regulators and through them, the public, are demanding more accountability from our profession around the world. At a time of such diversity in many countries, Canada included, the need for national licensing examinations is increasing not decreasing.

In 1990, Canada’s MRAs requested that MCC become the first organization to implement a national clinical skills examination. The debate at that time was whether the examination should be at the end of medical school or after postgraduate education. Council concluded that the postgraduate education added a level of clinical ability that should be assessed if the examination was testing performance prior to entry into independent practice. This was seen as a necessary addition to the assessments of specialty abilities by the certifying Colleges.

In 2009, MCC initiated the Assessment Review Task Force (ARTF) which reviewed all aspects of the MCC activities. Focus groups were held with Faculties of Medicine and regulatory authorities across the country and medical educators were invited to provide opinion pieces. There was a split of opinion amongst some educators. A few wanted to move the clinical skills component of the MCC examination to the point of graduation from medical school. This, they argued, would allow graduates to enter their specialty area earlier. A larger number of educators argued that the examinations should remain in the postgraduate education time period to promote the retention of general competencies prior to developing specialty abilities. The regulators supported continuing the assessment at the end of the first postgraduate year. As a result, the ARTF committee elected to retain the current placement of the clinical skills assessment and all the recommendations from the ARTF (2011) were accepted unanimously by Council. These recommendations have provided the strategic direction for Council over the last five years.

Following an extensive practice analysis, the extent to which current MCC examinations fulfilled the ARTF vision was assessed. This practice analysis led to the development and adoption of a new MCCQE blueprint (or test specifications) which re-emphasizes the need to assess core generic skills expected of all physicians, irrespective of specialty. While not ignoring the expert physician role, its focus revisited the essential skills of communication, collaboration and concerns about patient safety. The new blueprint also recognizes that the current MCCQE Part I and II will need to be supplemented by ancillary longitudinal in-practice assessment data points to be collected, both at the undergraduate and postgraduate levels. The impact of the new blueprint cannot be underestimated. This is especially true for the MCCQE Part II as the test committee is revisiting content, station formats and scoring approaches with the goal of moving towards more contexts reflecting issues of patient safety and the complexity of care while continuing valid assessment of post graduate trainees. The changes being piloted now will mean less emphasis on basic history taking and physical examination skills and far more focus on assessing and managing complex patient scenarios and professional interactions with colleagues and allied health professionals in the context of providing clinical care. The full extent of this work will only be truly seen in 2018.

Point in time examinations will remain a part of the core assessment of any candidate based on previous validity evidence. In one of the rare outcomes reviews of physician performance related to examinations Tamblyn et al.^2^ demonstrated that performance on the MCC’s examinations predicts performance out in practice in such categories as health promotion, illness prevention and the use of practice guidelines. Tamblyn et al.^3^ also looked at physician performance up to ten years in practice using the complaints process in Quebec and Ontario. She demonstrated that “scores achieved in patient-physician communication and clinical decision making on the MCCQE Part II predicted complaints to medical regulatory authorities.”

More recent studies by Pugh et al.^4^ demonstrated that postgraduate education, at least in the first year, does improve OSCE performance dramatically and that this performance also predicts performance on Royal College internal medicine examinations.

In an era of increasing public accountability, the profession should not be considering eliminating an examination that holds physicians to widely accepted pan-Canadian standards. Rather, it is time to use the outcome studies by Tamblyn et al. to identify those physicians at risk for future problems in practice and provide follow-up, mentoring and support.

MCC examinations are accepted as a critical requirement for Canadian licensure by all MRAs. The primary purpose of the examinations has always been to measure the required knowledge, skills and behaviours that are core to being an independent physician. The MCC examinations have been subjected to rare outcomes studies and, unlike many examinations offered at different stages in medical education, have been shown to predict performance in practice. The recent and extensive practice analysis process was undertaken to ensure that the examinations offered by the MCC are even more relevant to the modern day practice of medicine.
